# On the Critical Issues in Temozolomide Research in Glioblastoma: Clinically Relevant Concentrations and MGMT-independent Resistance

**DOI:** 10.3390/biomedicines7040092

**Published:** 2019-11-27

**Authors:** Aleksei A. Stepanenko, Vladimir P. Chekhonin

**Affiliations:** 1 Department of Fundamental and Applied Neurobiology, V.P. Serbsky National Medical Research Center for Psychiatry and Narcology, The Ministry of Health of the Russian Federation, Kropotkinsky Lane 23, 119034 Moscow, Russia; chekhoninnew@yandex.ru; 2 Department of Medical Nanobiotechnologies, Medico-Biological Faculty, N.I. Pirogov Russian National Research Medical University, The Ministry of Health of the Russian Federation, Ostrovitianov Str. 1, 117997 Moscow, Russia

**Keywords:** Glioblastoma, glioma, temozolomide resistance, O^6^-methylguanine-DNA methyltransferase

## Abstract

The current standard first-line treatment for adult patients with newly diagnosed glioblastoma includes concurrent radiotherapy and daily oral temozolomide (TMZ), followed by adjuvant TMZ. As a prodrug, TMZ undergoes spontaneous hydrolysis generating a methylating agent. O^6^-methylguanine is considered the most preponderant toxic damage mechanism at therapeutically relevant TMZ doses, whereas *MGMT*, which encodes the O^6^-methylguanine-DNA methyltransferase DNA repair enzyme, is the most relevant resistance mechanism. Speculations on clinically relevant TMZ concentrations, cytotoxic and cytostatic effects of TMZ, and resistance mechanisms exist in the literature. Here, we raise the following principal issues: What are the clinically relevant TMZ concentrations in glioma patients, and which TMZ-induced molecular lesion(s) and corresponding resistance mechanism(s) are important for TMZ therapeutic effects at clinically relevant concentrations? According to clinical data from patients with glioblastoma, the mean peak TMZ concentrations in the peritumoral tissue might be much lower (around 5 µM) than usually used in in vitro research, and may represent only 20% of systemic drug levels. According to in vitro reports, single-dose TMZ at concentrations around 5 µM have minimal, if any, effect on apoptosis and/or senescence of glioblastoma cell lines. However, the clinically relevant concentrations of TMZ are sufficient to radiosensitize both MGMT-positive and -negative cell lines in vitro. It is speculated that a single DNA repair protein, MGMT, is highly efficient in protecting cells against TMZ toxicity. However, an endogenous level of MGMT protein expression is not universally correlated with TMZ responsiveness, and MGMT-independent mechanisms of TMZ resistance exist.

The current standard first-line treatment for adult patients with newly diagnosed glioblastoma includes concurrent radiotherapy and daily oral temozolomide (TMZ, 75 mg/m^2^ body surface area (BSA)/day), followed by adjuvant TMZ (150–200 mg/m^2^ BSA/day). As a prodrug, TMZ undergoes spontaneous hydrolysis generating a methylating agent. O^6^-methylguanine (O^6^MeG) is considered the most preponderant toxic damage mediator at therapeutically relevant TMZ doses, whereas *MGMT*, which encodes DNA-repair O^6^-methylguanine-DNA methyltransferase that removes alkyl groups from the O^6^ position of guanine, constitutes the most relevant resistance mechanism [1]. In a recent review [2], and in a comment on this review [3], both published in *Biomedicines*, Strobel and colleagues on the one hand and Kaina on the other hand, came to opposite conclusions on the cytotoxic and cytostatic effects of TMZ. Strobel et al. deduced that “cell death… is usually only produced in experimental systems with non-physiologically high concentrations, often in the range of 100 µM TMZ (refs) up to 1000 and 4000 µM [refs.], while models predict a peak concentration in the tumor in the range of 14.95–34.54 µM (ref)” and speculate that “Possibly, TMZ should be considered primarily cytostatic and senescence-inducing and not cytotoxic and apoptosis-inducing (ref)…” [2]. In contrast, Kaina discusses that TMZ induces autophagy, senescence, and apoptosis in glioblastoma cell lines with the clinically relevant TMZ doses (up to 25–50 μM) [3]. Referring to his own research, Kaina states: “TMZ is clearly a cytotoxic drug, as cells following treatment die by apoptosis even at low dose levels (refs)” [3]. In light of these opposing statements, the principal issue is what the clinically relevant TMZ concentrations in glioma patients are.

Rosso et al. characterized TMZ distributions in brain tissue based on [methyl-^11^C]TMZ positron emission tomography (PET) data from patients with recurrent glioma (*n* = 7) receiving 75–200 mg/m^2^ BSA/d, and predicted the peak brain tumor TMZ concentrations ranging from 2.9 to 6.7 µg/mL (14.94–34.51 µM) for different TMZ dosing regimens [4]. This study has been frequently cited in the literature when referring to “the clinically relevant TMZ concentrations”. However, other reports provide evidence for lower TMZ concentrations in the peritumoral tissue or cerebrospinal fluid. Ostermann et al. determined that the cerebrospinal fluid TMZ levels ranged 0.16–1.93 µg/mL (0.82–9.94 µM) in samples collected at occasional time points from patients receiving the daily TMZ dose of 75 or 200 mg/m^2^ BSA [5]. Portnow et al. used an intracerebral microdialysis catheter placed in the peritumoral tissue to determine TMZ concentrations by tandem mass spectrometry. Patients (*n* = 7) received a single oral dose of TMZ (150 mg/m^2^ BSA) on the first postoperative day and serial samples were collected over 24 hours. The mean peak TMZ concentration in the peritumoral tissue was 0.6 ± 0.3 μg/mL (1.55–4.64 µM) [6]. Recently, these data have been independently confirmed in a study of similar design. The samples from five patients with recurrent glioblastoma who received oral TMZ (150 mg/m^2^ BSA) were collected by intracerebral microdialysis catheters on post-operative day one and analyzed by tandem mass spectrometry. The TMZ concentration in the peritumoral tissue was 0.55 ± 0.26 µg/mL (1.49–4.17 µM) [7]. Altogether, it appears that the clinically relevant TMZ concentrations might be around 5 µM, and TMZ levels in the brain are only 20% of systemic drug levels [5,6,7]. Therefore, all in vitro studies should evaluate TMZ effects using these low concentrations. According to in vitro reports, single-dose TMZ at concentrations around 5 µM have minimal, if any, effect on apoptosis and/or senescence of glioblastoma cell lines (e.g., U87, LN229, LN308, U138, SNB19, A1235, SF767, T98G, and MR4) [8,9,10] and TMZ might be considered a cytostatic rather than cytotoxic drug when applied at single-dose low concentrations, as suggested by Strobel and colleagues [2]. However, low concentrations of TMZ (5–10 µM) were sufficient to radiosensitize both MGMT-positive and negative cell lines in vitro [10,11]. Thus, at clinically relevant low concentrations, TMZ in combination with radiotherapy, but not TMZ alone, is effective in inducing massive cell death. Nevertheless, the surviving glioma cells with intrinsic or acquired resistance during the course of therapy give rise to recurrence in in vivo models, and in the clinical setting. Hence, another important issue that should be addressed is which TMZ-induced lesion(s) and which corresponding resistance mechanism(s) are important for achieving therapeutic effects at clinically relevant low concentrations of TMZ. 

In his comments, Kaina concluded: “Taken together, the available data show undoubtedly that the TMZ-induced DNA adduct O^6^MeG is a highly cytotoxic, genotoxic, recombinogenic and DNA damage response (DDR)-activating lesion. The data demonstrate at the same time that a single DNA repair protein, MGMT, is highly efficient in protecting against all these effects (ref). Importantly, already at low dose levels, O^6^MeG is a powerful activator of the apoptosis pathway (refs) …” [3]. *MGMT* promoter methylation is a well-established prognostic factor on multivariate analysis. In phase III clinical trials, *MGMT* promoter methylation has been associated with longer overall survival in patients with glioblastoma who received TMZ in addition to radiotherapy [12], and was included in an independently validated nomogram for individualized estimation of survival among patients with newly diagnosed glioblastoma [13]. However, the predictive value of *MGMT* promoter methylation for TMZ responsiveness is not unequivocally reproducible across studies. When *MGMT* promoter methylation status in glioblastoma samples classified as classical (*n* = 96), proneural (*n* = 66), neural (*n* = 55), and mesenchymal (*n* = 104), was correlated with outcome, The Cancer Genome Atlas (TCGA) Research Network summarized: “*MGMT* DNA methylation may be a predictive biomarker for treatment response only in classical subtype glioblastoma” [14]. The diagnostic method of choice (e.g., pyrosequencing or quantitative reverse transcription PCR), variable contamination of tumor samples by non-neoplastic cells, the quality of DNA extracted from fixed and embedded tissue, the technical cut-off value defined for differentiating between *MGMT*-methylated and *MGMT*-unmethylated samples and other analytical variables may affect data interpretation, highlighting the need for the development of guidelines, and internal and external quality control measures [15]. It may seem that the level of MGMT protein expression should predict TMZ sensitivity more accurately. Unfortunately, this is also not the case. Meyer et al. demonstrated that single cell-derived clones of human glioblastoma samples exhibited heterogeneous TMZ responsiveness (treated at a dose range of 0.39–100 μM), which was uncorrelated with *MGMT* promoter methylation and MGMT protein expression. The authors concluded: “Importantly, a clonal analysis of the standard glioblastoma biomarker MGMT did not correlate with TMZ responsiveness, suggesting that new biomarkers of drug responsiveness are sorely needed…” [16]. Based on evaluation of relationships between *MGMT* promoter methylation, MGMT protein expression, and TMZ responsiveness in 13 established intracranial tumor xenografts in mice, Kitange et al. concluded that “MGMT protein expression is insufficient to confidently predict tumor TMZ responsiveness in individual patients”, since tumors with low but detectable MGMT expression showed remarkable variability in responsiveness [17]. In this study, the authors calculated that TMZ administered to mice by oral gavage at 66 mg/kg for five days results in a drug exposure that is equivalent to that obtained in humans during an adjuvant dosing regimen of 200 mg/m^2^ BSA × 5 days. On a panel of 15 diffuse large B-cell lymphoma cell lines, Leshchenko et al. found no correlation between TMZ sensitivity (when treated with 25 to 2500 μM) and *MGMT* promoter methylation, mRNA or MGMT protein expression, although concordance between *MGMT* methylation and expression was reported [18]. In addition, there are other reports showing that cell lines did not express MGMT protein yet were resistant to TMZ at clinically relevant low doses or supraphysiological or non-physiological concentrations, and vice versa, cell lines expressing MGMT protein demonstrated sensitivity to TMZ. Altogether, these studies clearly point out that endogenous levels of MGMT protein expression are not universally correlated with TMZ responsiveness, and that MGMT-independent mechanisms of TMZ resistance do exist. In support of this notion, MGMT-negative cells acquire TMZ resistance to supraphysiological and non-physiological concentrations in a short time period ([19] and refs. herein). Acquired resistance to high doses of TMZ is associated with significant changes in the genome, in DNA methylation, the transcriptome, proteome, kinome, and metabolome ([19] and refs. herein). In addition to MGMT, other DNA repair pathways and proteins not directly related to repair systems were found to modulate cellular sensitivity to TMZ (refs. and suppl. data in [19]). Currently, it remains uncertain which MGMT-independent resistance mechanisms described in the literature do significantly operate at clinically relevant low TMZ concentrations.

In the end, it should be emphasized that the high rate of failure in glioblastoma clinical trials assessing targeted drugs [12], as well as pharmaceutically important features of TMZ such as acid stability, oral bioavailability, absorption, hydrolysis kinetics, metabolic half-life, biodistribution, and blood-brain barrier penetration [20], rather than the satisfactory therapeutic efficiency cemented TMZ prescription for glioblastoma patients worldwide. Irrespective of cytostatic and/or cytotoxic effects of TMZ at clinically relevant low concentrations in in vitro and in vivo models, its therapeutic efficiency even in patients with *MGMT*-methylated tumors is limited, clearly suggesting that alternative or additional therapeutic approaches are urgently needed [12]. 
